# Downstream Processing of Amorphous and Co-Amorphous Olanzapine Powder Blends

**DOI:** 10.3390/pharmaceutics14081535

**Published:** 2022-07-23

**Authors:** Nuno F. da Costa, Rolf Daniels, Ana I. Fernandes, João F. Pinto

**Affiliations:** 1 iMed.ULisboa—Research Institute for Medicines, Faculdade de Farmácia, Universidade de Lisboa, Av. Prof. Gama Pinto, 1649-003 Lisboa, Portugal; nparferreira@gmail.com (N.F.d.C.); jfpinto@ff.ul.pt (J.F.P.); 2 Department of Pharmaceutical Technology, Eberhard Karls University, Auf der Morgenstelle 8, D-72076 Tuebingen, Germany; rolf.daniels@uni-tuebingen.de; 3 CiiEM—Interdisciplinary Research Center Egas Moniz, Instituto Universitário Egas Moniz, Monte de Caparica, 2829-511 Caparica, Portugal

**Keywords:** (co-)amorphous, cohesiveness, compressibility, flowability, olanzapine, tablet

## Abstract

The work evaluates the stability of amorphous and co-amorphous olanzapine (OLZ) in tablets manufactured by direct compression. The flowability and the compressibility of amorphous and co-amorphous OLZ with saccharin (SAC) and the properties of the tablets obtained were measured and compared to those of tablets made with crystalline OLZ. The flowability of the amorphous and mostly of the co-amorphous OLZ powders decreased in comparison with the crystalline OLZ due to the higher cohesiveness of the former materials. The stability of the amorphous and co-amorphous OLZ prior to and after tableting was monitored by XRPD, FTIR, and NIR spectroscopies. Tablets presented long-lasting amorphous OLZ with enhanced water solubility, but the release rate of the drug decreased in comparison with tablets containing crystalline OLZ. In physical mixtures made of crystalline OLZ and SAC, an extent of amorphization of approximately 20% was accomplished through the application of compaction pressures and dwell times of 155 MPa and 5 min, respectively. The work highlighted the stability of amorphous and co-amorphous OLZ during tableting and the positive effect of compaction pressure on the formation of co-amorphous OLZ, providing an expedited amorphization technique, given that the process development-associated hurdles were overcome.

## 1. Introduction

Consideration of drug bioavailability, defined as the fraction of drug that reaches the systemic circulation and thus becomes available at the site of action [1], is paramount in pharmaceutics. Currently, only 5–10% of new drug candidates possess the desired solubility and permeability for acceptable oral bioavailability [2]. In this respect, bioavailability concerns are mostly associated with insufficient solubility in water rather than with the poor permeability of drugs across membranes [2]. The increasing number of drug entities presenting reduced water solubility may be explained by the development of high molecular weight compounds capable of establishing multiple intermolecular hydrogen bonds [3]. As a consequence of the drug’s poor bioavailability, higher doses are required to reach the effective plasma concentration required to promote the therapeutic effect. This results in higher costs for both manufacturer and patient (e.g., increased dose loads to compensate for poor absorption) and amplifies side effects during treatment (e.g., diarrhea, nausea, abdominal discomfort, vomiting, or abdominal pain) [4,5].

Substantial interest has thus been provided to the study of novel approaches aimed at enhancing the bioavailability of drugs, especially those capable of increasing drug solubility [6,7,8,9,10,11]. Among these, the preparation and stabilization of amorphous drugs using low molecular weight compounds—producing co-amorphous systems (CAMs)—has been considered a viable strategy, resulting in a suitable compromise between solubility improvement and stability in the amorphous form [8,12,13,14,15]. Löbmann et al. [16] showed that co-amorphization could be used to enhance the stability of pure amorphous indomethacin or carbamazepine. In this regard, the authors demonstrated that the CAMs containing indomethacin and carbamazepine were stable for more than 6 months at room temperature and dry conditions, while the corresponding pure amorphous drugs recrystallized shortly after manufacture. Simultaneously, these CAMs have shown a significant increase in dissolution rate compared to crystalline and pure amorphous drugs [16]. The disordering of crystalline into amorphous materials can be accomplished using different preparation methodologies that are based on thermodynamics or kinetic mechanisms [17,18]. Quench cooling, solvent evaporation, or ball milling are well-accepted methods used to prepare amorphous and CAM materials. Concomitantly, other unit operations, such as tableting, have also proved to be effective in the preparation of amorphous materials [19,20]. To date, a notable endeavor has been put into the development of CAMs, namely on the selection of the co-formers [13,21,22,23], the adequate drug: co-former molar ratio [24] and CAM preparation methods [11,25,26]. However, the formulation of CAMs in oral solid dosage forms (e.g., tablets) has been barely described [27,28,29]. As an example, Lenz et al. [28] have shown that the tableting of an indomethacin: arginine CAM salt has not resulted in the recrystallization of the compounds over a broad range of compaction pressures applied to the systems.

Olanzapine (OLZ), the model drug used in this study, is a benzodiazepine commonly used in the treatment of schizophrenia, acute manic episodes, and bipolar disorder due to the reduced side effects (e.g., extrapyramidal effects) and high therapeutic efficacy [30,31]. OLZ is a Biopharmaceutical Classification System class II drug with a bioavailability of 60% as a result of extensive hepatic metabolism and poor solubility in water [31]. Several strategies to increase drug solubility and dissolution rate, as a way of increasing the amount of OLZ made available at the action site, have been attempted [32,33,34]. Previously, the amorphization of OLZ was shown to promote a 4.6-fold enhancement in the water solubility of the drug compared to its crystalline counterpart (202.1 mg/L versus 47.7 mg/L, at 37 °C) [19,21]. However, the instability associated with the amorphous OLZ resulted in the recrystallization of the amorphous form back to its crystalline counterpart in less than 4 months, at 23 °C and 65% of relative humidity. On the contrary, co-amorphization of OLZ with saccharin (SAC) increased the stability of the CAM prepared (>4 months, at 23 °C and 65% of relative humidity) [19].

Direct compression of powder blends is a valuable approach for tablet production, commonly used by the pharmaceutical industry, as it allows the manufacture of tablets in a simple, cost-effective, and expedite manner. However, the inappropriate flowability and compressibility-related attributes of the starting powder blends can compromise the processability of formulations [35,36]. The measurement of the flowability of mixtures can be used to anticipate the filling behavior of the die cavities of the tableting machine [37], which in turn impacts the quality attributes of drug products (e.g., weight and dose uniformities) [37,38]. Compendial methods, such as the angle of repose, Hausner ratio, or Carr’s index, have been employed for many years to describe both flowability and compressibility [39]. Recently, the development of new instrumented techniques enabled the determination of the cohesiveness of mixtures and elucidated its effect on flowability [40] and compressibility [41].

Based on the rheological properties of amorphous and co-amorphous OLZ powder blends, this work aimed to evaluate their suitability for the manufacture of tablets by direct compression while maintaining their physical stability. Furthermore, the effect of compression pressure and dwell time during tableting were also assessed.

## 2. Materials and Methods

### 2.1. Materials

OLZ was a gift from Rampex Labs Pvt. Ltd. (Telangana, India). SAC (Sigma-Aldrich, Steinheim, Germany) was used as the co-former and dichloromethane (Biochem Chemopharma, Cosne sur Loire, France) as the solvent for the preparation of OLZ-CAM. Liquid nitrogen (Air Liquide, Lisbon, Portugal) was used to prepare amorphous OLZ. Tablets were manufactured from blends of OLZ and SAC, dibasic calcium phosphate anhydrous (DI-CAFOS^®^ A60, a gift from Budenheim, Budenheim, Germany), microcrystalline cellulose (Avicel PH-101, FMC Corp., Cork, Ireland), polyvinylpyrrolidone (K25, BASF, Ludwigshafen, Germany) and croscarmellose sodium (JRS Pharma, Rosenberg, Germany). Demineralized water (Destillo 2, Herco, Freiberg am Neckar, Germany), sodium hydroxide (Eka, Marietta, GA, USA), and potassium phosphate monobasic (Carlo Erba Reagents, Val de Reuil, France) were used to prepare the phosphate buffer (pH 8.0) for the dissolution studies. Marketed tablets of OLZ (15 mg, Generis Farmacêutica, S.A., Amadora, Portugal) were used as controls in the dissolution studies. A desiccator containing magnesium nitrate (53% RH, ThermoFisher GmbH, Kandel, Germany) was used to store amorphous OLZ and OLZ-CAM.

### 2.2. Methods

#### 2.2.1. Preparation of Amorphous and Co-Amorphous Olanzapine

Preparation of amorphous OLZ: Pure OLZ was heated up to 200 °C to ensure the complete melting of the drug (melting point = 194 °C [42]). Afterward, the melt was rapidly quenched using liquid nitrogen to preserve the molecular conformation of the liquid state material.

Preparation of co-amorphous OLZ: OLZ and SAC, in a 1:1 molar ratio, were dissolved in pure dichloromethane prior to the evaporation of the solvent under vacuum (45 °C, 650 mbar, R-100, Buchi Rotavapor, Flawil, Switzerland). The CAM produced was left under vacuum for 24 h for the complete removal of the solvent. All the materials were stored in desiccators containing magnesium nitrate (53% RH) for the rest of the studies.

#### 2.2.2. Characterization of Pure Amorphous and Co-Amorphous Olanzapine

Prior to the characterization and processing of the crystalline, amorphous OLZ and OLZ-CAM, samples were gently milled using a mortar and pestle and sieved through a 180 µm mesh to ensure a uniform particle size (125–180 µm fraction). Amorphous OLZ and OLZ-CAM were analyzed immediately after preparation.

X-ray Powder Diffraction (XRPD): XRPD analysis was conducted, in duplicate, using a PANalytical X-ray diffractometer (X’Pert PRO, PANalytical, Almelo, the Netherlands). A CuKα source of radiation (λ = 1.54 Å) at 40 kV and 30 mA was used to analyze the samples using a step size of 0.017° 2θ and a counting time of 19.685 s within the range 7 to 35° 2θ.

Differential Scanning Calorimetry (DSC): Thermal characterization of pure crystalline, pure amorphous OLZ, and OLZ-CAM was performed in a TA calorimeter (Q200, TA Instruments, New Castle, USA). Duplicates samples (approximately 5 mg) were placed in aluminum pans (THEPRO GbR, Heinsberg, Germany) and then run using the modulated DSC program from −40 to 240 °C, a nitrogen gas flow of 50 mL/min, a heating rate of 2 °C/min, and an amplitude of 0.318 °C for a period of 60 s. The data obtained were analyzed using proprietary software (Universal Analysis 2000, version 4.7A, 2009, TA Instruments, New Castle, DE, USA), and the glass transition temperature (Tg) was calculated as the midpoint of the thermal event in the thermograms.

Assessment of flowability (rheometric analysis of blends): powder flow measurements (n = 3) were performed using a TA.XT Plus Texture Analyzer equipped with a powder flow analyzer attachment (TA.XT Plus, Stable Micro Systems, Surrey, UK). A total of 30 g of material were placed in the equipment vessel (120 mm height and 50 mm internal diameter glass container,) to evaluate the cohesiveness and the cake properties of the materials. Before each test, the samples were conditioned using a downward movement of the blade (50 mm/s) and an upward movement (50 mm/s) to remove any variability in the results due to a non-uniform filling. The cohesion test involved the downward movement of the blade at an axial tip rate of 20 mm/s and an upward movement at 75 mm/s (i.e., rotational speed of approximately 20 rpm). The cohesion index was found as the ratio between the work required to move up the blade (cohesion coefficient, Equation (1)) and the weight of the sample [43].

(1) $$\mathrm{Cohesion}\;\mathrm{Index}\;\left(\mathrm{mm}\right)=\frac{\mathrm{Cohesion}\;\mathrm{Coefficient}\;\left(g\cdot {\mathrm{mm}}^{-1}\right)}{\mathrm{Weight}\;\left(g\right)}$$

For the measurements of caking, 5 compaction cycles were applied to the samples. This was achieved by moving the blade down (20 mm/s, rotational speed varying from 3 to 22 rpm) through the sample until a compaction force of 5 N was reached. After 5 cycles, the blade overpassed the cake built, and the force required to break down the cake was recorded (cake strength, in g). The analysis of data was performed using proprietary software (Exponent, version 6.1.11.0, 2016, Stable Micro Systems, Surrey, UK).

Angle of Repose: Samples (20 g) were placed in a funnel (Ø = 13 mm) located at a predefined height above a plate [44]. The discharge of each sample through the funnel resulted in the formation of a cone with a round basis. The angle of repose was calculated by measuring the diameter (D, in Equation (2)) and the height (H, in Equation (2)) of the cone (Equation (2)).

(2) $$\mathrm{Angle}\;\mathrm{of}\;\mathrm{repose}\;\left({}^{\circ}\right){=\tan}^{-1}(\frac{2\;\times \;H}{D})$$

Density: The true densities of raw materials, powdered blends, and tablets were measured (n = 3) by pycnometry (helium at 18.5 psig, AccuPyc 1330, Micromeritics, GA, USA) at room temperature (25 °C).

Bulk and Tapped Density: A 50 cm^3^ cylinder was filled with the sample, and the unconfined bulk density was determined. Then, the cylinder was tapped (density apparatus Stampfvolumeter STAV 2003, Jel, Ludwigshafen am Rhein, Germany) for 1250 taps for a constant volume, which was recorded (n = 3) prior to the calculation of the tap or confined density of the sample.

#### 2.2.3. Formulation of Tablets

The crystalline, amorphous, and OLZ-CAM were formulated to allow further processing into tablets (Table 1). Formulation A considered the use of pure crystalline and pure amorphous OLZ, while formulation B considered the use of the physical mixture with both crystalline OLZ and SAC and the OLZ-CAM entity thus produced.

#### 2.2.4. Blending

Mixtures (60 g) were prepared according to either formulation A or B (Table 1) and blended in a rotating mixer (225 cm^3^, 60 rpm, Fisher-Kendall 12-811, Fisher-Kendall Scientific Co., Pittsburgh, PA, USA) for 10 min.

#### 2.2.5. Tableting

Powdered mixtures (66.5 mg) of formulation A (either pure crystalline or pure amorphous OLZ) or formulation B (physical mixture of crystalline OLZ:SAC or OLZ-CAM)—equivalent to 20 mg of OLZ—were accurately weighted and directly compacted at 25, 90 and 155 MPa using a universal testing machine (Lloyd Instruments, LR50K Plus, Largo, FL, USA). Flat punches (5.0 mm Ø) were used to compact the mixtures at a predefined compression rate of 10 mm/min. The work of compaction and the ejection force were found from the data in the graphical representations of the punch displacement vs. the load applied to the samples (Nexygen Plus, version 3.0, 2013, Largo, FL, USA).

For the quantification of the fraction of amorphous OLZ, tablets of 300 mg were manufactured using 10.0 mm Ø flat punches to mimic the conditions described previously [19]. Tablets were compacted at 25, 90, and 155 MPa, at a predefined compaction rate of 10 mm/min for different dwell times (0, 2, and 5 min).

#### 2.2.6. Quantification of the Fraction of Amorphous and Co-Amorphous Olanzapine

Regression models previously developed [19] were used to quantify the fraction of amorphous OLZ and OLZ-CAM present in physical mixtures and tablets, based on the data provided by the near-infrared spectroscopy (NIR) (in the wavenumber region between 6094 and 5577 cm^−1^), Fourier-transform infrared spectroscopy (FTIR) (1620–1500 cm^−1^) and XRPD (20.7–21.2° 2θ). Briefly, the models proved to be linear and accurate for the detection of the fraction of amorphous and crystalline OLZ, as the error of prediction for external samples was as low as 0.9%, 4.2%, and 2.9% for NIR, FTIR, and XRPD characterization methods, respectively [19].

Near-Infrared Spectroscopy: NIR spectroscopy was conducted on an ABB spectrometer (TLA 2000, ABB, Québec, QC, Canada) fitted with an indium-gallium-arsenide detector. Then, samples (n = 3) were scanned 32 times each within the wavenumber range 10,000 to 4000 cm^−1^, at a resolution of 8 cm^−1^, using polytetrafluoroethylene (PTFE) as background (SKG8613G, ABB, Québec, QC, Canada). Data were analyzed with the Spectragryph software (Spectragryph, version 1.2.13, 2019, Oberstdorf, Germany).

Fourier-Transform Infrared Spectroscopy: FTIR spectroscopy was conducted on a Bruker spectrometer (Alpha II, Bruker, MA, USA) connected to a diamond ATR accessory (Platinum ATR, Bruker, MA, USA). Samples (n = 3) were then scanned 24 times over the wavenumber range 4000–525 cm^−1^ at a resolution of 4 cm^−1^. Data analysis and treatment were conducted using the Spectragryph software (Spectragryph, version 1.2.13, 2019, Oberstdorf, Germany).

#### 2.2.7. Stability Studies

Stability studies of tablets were run for 3 years at 23 °C/65% of relative humidity to mimic shelf storage conditions. At the end of the experiment, samples were analyzed by XRPD to evaluate the recrystallization of OLZ-CAM.

#### 2.2.8. Characterization of Blends and Tablets

Characterization of tablets (n = 3) was performed 1 day after manufacture to allow the relaxation of tablets at room temperature.

Tensile Strength: A TA.XT Plus Texture Analyzer fitted with a cylinder probe (TA.XT Plus, Stable Micro Systems, Surrey, UK) was used to measure the crushing force of tablets at a testing speed of 100 mm/min for crushing forces below 50 N. When the force required to crush the tablets was higher than 50 N, an Erweka apparatus (TBH 20, Erweka, Heusenstamm, Germany) was used. The tensile strength was calculated from the crushing force according to Equation (3) [45].

(3) $$\mathrm{Tensile}\;\mathrm{Strength}=\frac{2\times \;\mathrm{Crushing}\;\mathrm{Force}}{\pi \;\times \;\mathrm{Diameter}\;\times \;\mathrm{Thickness}}$$

Disintegration Time: The disintegration time of tablets was determined in a disintegration apparatus (ZT3, Erweka, Heusenstamm, Germany) [44] containing demineralized water at a predefined temperature of 37.0 ± 0.5 °C.

Dissolution Studies: Dissolution studies (n = 3) were conducted on tablets and powdered blends of materials in a dissolution apparatus (AT7, Sotax, Aesch, Switzerland) considering the paddle method (at 100 rpm) [44]. A total of 300 mL of phosphate buffer pH 8.0 (at which the drug is predominantly unionized) was used as the dissolution medium (37 ± 0.5 °C) to impose the saturation of OLZ. As controls, 2 marketed tablets containing 15 mg of OLZ were placed in 450 mL of phosphate buffer pH 8.0. Aliquots (2 mL) were taken at predefined times (0, 2, 5, 10, 15, 30, 60, 90, 120, and 150 min), and fresh dissolution medium was added to maintain the volume constant (300 mL). Samples were filtered through a 0.22 µm MCE filter (Merck, MA, USA) and analyzed by UV spectrophotometry (λ = 254 nm, U-1900, Hitachi, Tokyo, Japan).

#### 2.2.9. Statistical Analysis

Statistical data analysis was conducted using the independent-samples t-test, assuming that the variances were unequal (SPSS Statistics, version 27.0.1.0, 2020, IBM, Armonk, NY, USA). Differences were considered statistically significant for *p*-values < 0.05 (95% confidence level).

## 3. Results and Discussion

### 3.1. Assessment of the Amorphization and Co-Amorphization of Olanzapine

OLZ is an antipsychotic drug that presents a solubility in water of 48 mg/L (at 37 °C) [19]. Calorimetric studies have shown a melting temperature and enthalpy of 192.4 °C and 109.8 J/g, respectively, whereas XRPD measurements revealed the presence of sharp and intense peaks at 8.6, 14.5, 17.0, 19.8, 21.0, 21.5, 22.3 and 23.9° 2θ which were attributed to the polymorphic anhydrous form I of OLZ [42,46].

**Figure 1:**
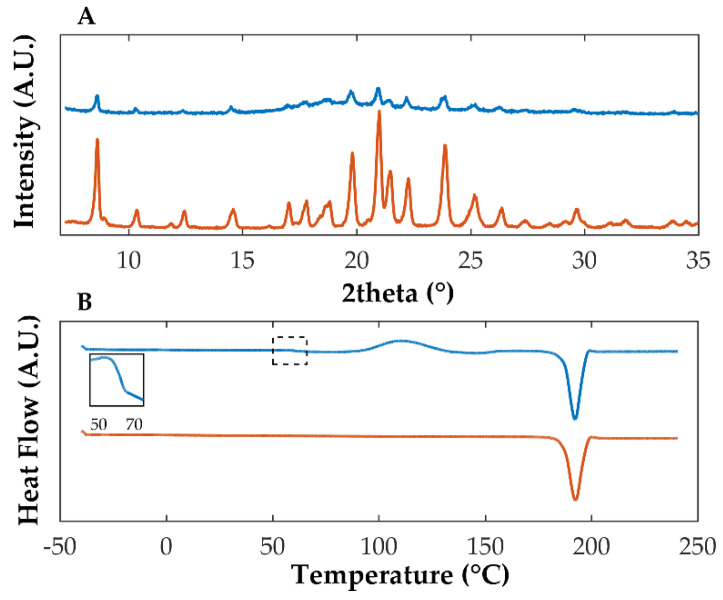
Diffractograms (**A**) and thermograms (**B**) of quench cooled (blue) and crystalline (orange) OLZ. The insert in thermogram B enlarges the glass transition region of amorphous OLZ.

As shown elsewhere [19], when quench cooled, OLZ was partially converted into its amorphous counterpart. This was ascertained by the intensity reduction in the characteristic XRPD peaks and the presence of a halo pattern in the diffractogram (Figure 1A). The appearance of the halo pattern in diffractograms has been considered for the confirmation of the production of amorphous materials as these scatter the X-rays in different directions due to the absence of long-range molecular order [47]. Concomitantly, thermograms (Figure 1B) presented a Tg at 67 °C, indicating the presence of amorphous OLZ before recrystallization at higher temperatures (exothermic event at 103.3 °C—onset temperature of recrystallization) and melting of the recrystallized OLZ (endothermic event at 192.5 °C).

Even though the amorphization of the drug was incomplete, a 3.5-fold solubility enhancement (compared to the polymorphic form I of OLZ, 141.4 mg/L versus 40.6 mg/L, phosphate buffer pH 8.0, at 37 °C) was achieved, thus anticipating a higher bioavailability of the drug.

**Figure 2:**
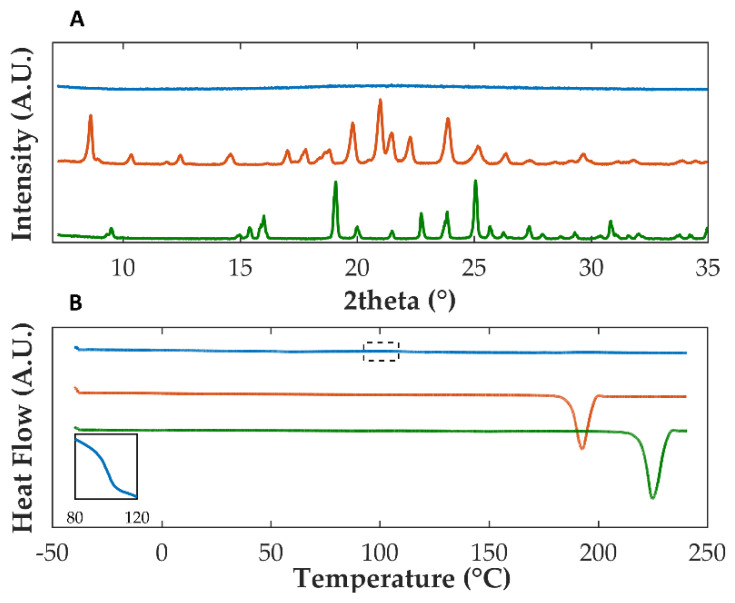
Diffractograms (**A**) and thermograms (**B**) of co-amorphous OLZ (blue), pure crystalline OLZ (orange), and pure crystalline SAC (green). The insert in thermogram B enlarges the glass transition region of co-amorphous OLZ.

Previously, sulfonic acid derivatives demonstrated their ability to stabilize OLZ in the amorphous form [48]. Among the co-formers considered, SAC allowed the production (solvent evaporation preparation technique) of the CAM with the highest solubility increase (145-fold enhancement in comparison with OLZ form I, 5895.6 mg/L versus 40.6 mg/L, phosphate buffer pH 8.0, at 37 °C) and the longest stability period (>24 weeks at 75% RH/25 °C). Diffractograms of solvent evaporated OLZ:SAC presented a halo pattern, and the peaks attributable to crystalline structures within the materials were absent, which indicated the (co-)amorphization of both compounds (Figure 2A). Concomitantly, a unique Tg was found at 100.8 °C, suggesting the miscibility and amorphization of both OLZ and SAC (Figure 2B) and the absence of thermal events related to the melting of crystalline structures, in line with the data gathered by XRPD.

### 3.2. Rheological Characterization of Crystalline, Amorphous, and Co-Amorphous Olanzapine

The assessment of the rheological properties of powders is of great concern for the downstream processing of crystalline and amorphous materials [43,49]. Particularly, the flowability, agglomeration, and compressibility of powders can seriously compromise the handling, processing, and quality attributes of the final products [50].

Powder cohesiveness is related to the tendency for particles to cling together and form agglomerates. Amorphization and co-amorphization of OLZ increased the cohesiveness of the powdered systems. Co-amorphization of OLZ, in particular, resulted in the preparation of a powdered system, which exhibited an approximately 2-fold increase in the cohesion index compared to the physical mixture of crystalline OLZ and SAC (25.2 and 12.7 for CAM and crystalline OLZ:SAC, respectively; Table 2). This greater tendency for particles to cling together may be justified by the higher surface free energy of amorphous and CAM materials, in contrast with their crystalline counterpart. In a previous work, Ojarinta et al. [29] also observed that ibuprofen-arginine or indomethacin-arginine CAMs showed a higher cohesivity, which resulted in poor flowability, although the extent of the increase was not quantified.

Concomitantly, the higher cohesiveness of both amorphous OLZ and OLZ-CAM may also have promoted the superior caking ability of these materials, as reflected by the higher strength of the cake (Table 2). The force required to disrupt the cakes of amorphous OLZ and particularly OLZ-CAM allows us to infer the higher agglomeration ability of the respective powders during storage and handling with a significant negative impact on their processability, namely on discharging to or from the manufacturing equipment [51,52].

According to the angle of repose data, crystalline OLZ showed an angle of 51.7 ± 0.1°, reflecting its poor flowability (Table 2) [53]. The incorporation of SAC in a physical mixture with OLZ resulted in a slight decrease in the angle of repose value down to 51.0 ± 0.9° (*p* < 0.05). Amorphization, and particularly the co-amorphization of the drug, produced powdered systems with higher angles of repose (52.8 ± 0.3° and 54.6 ± 1.7° for amorphous and CAM, respectively) when compared to their crystalline counterpart. These results may indicate that the amorphous forms of OLZ possess additional difficulties to flow, a phenomenon that may be explained based on the higher cohesiveness and caking properties of pure amorphous OLZ and OLZ-CAM in comparison to the crystalline material. Herewith, it is worth mentioning that the particle size of all samples was kept constant (125–180 µm) to enable comparison between samples and to minimize the effect of particle size on the measurements.

Evaluation of the true densities of materials (pycnometry) revealed that crystalline OLZ, as a pure substance or in combination with SAC, presented a higher density (1.3053 ± 0.0022 g/cm^3^, pure form and 1.3897 ± 0.0017 g/cm^3^, in the physical mixture with SAC), than those of their amorphous counterparts (1.2764 ± 0.0014 g/cm^3^, quench cooled OLZ and 1.3501 ± 0.0058 g/cm^3^, for the CAM; Table 3). In crystalline solids, the higher density can be explained by the fact that molecules are more closely and regularly arranged due to the long-range molecular order. Inversely, amorphous materials present a short-range molecular order, preventing a closer arrangement and lacking the regular long-range organization of crystalline molecules [54]. These results were in line with previous observations of different fractions of amorphous polyethylene in which the 40% amorphous polyethylene showed a density of 0.910–0.925 g/cm^3^, significantly lower than the density obtained for polyethylene containing an amorphous fraction of 5% (0.940–0.970 g/cm^3^) [54].

Measurements of bulk and tap densities enabled the determination of Carr’s index (Table 3). Results showed that amorphous OLZ and OLZ-CAM exhibited higher compressibilities than their crystalline counterparts leading to higher consolidation abilities, anticipating the manufacture of tablets with higher tensile strengths. The high compressibility of powders (high values of Carr’s index) can be related to cohesive solids presenting poor flowabilities [39,55]. Carr’s index is thus aligned with the previous results (angle of repose, cohesion index, and cake strength) in what concerns the poor free-flowing behavior of amorphous OLZ and OLZ-CAM.

Overall, the results anticipate difficulties during tableting, particularly in the preparation of dosage forms containing potent active ingredients, for which mass and/or dosage uniformity are especially important. Thus, to enable the direct compression of tablets, it is of the utmost importance that the selection of excipients facilitates the flow of materials and, consequently, the preparation of dosage forms with the desired quality attributes.

### 3.3. Pressure-Induced Amorphization

The fraction of amorphous OLZ and OLZ-CAM present in blends and tablets were monitored using regression models previously established [19], which consider data gathered by NIR, FTIR, and XRPD. No solid-state conversions of crystalline and amorphous OLZ (both in the absence of SAC, formulation A) to other arrangements were observed as the result of the different compaction pressures applied during tableting. Similarly, no drug recrystallization in OLZ-CAM (formulation B) was detected upon exposure of powder beds to different compaction pressures. Since no recrystallization of OLZ-CAM was detected, tablets contained OLZ in a high energy form, anticipating improved solubility, dissolution rate, and bioavailability of OLZ.

**Figure 3:**
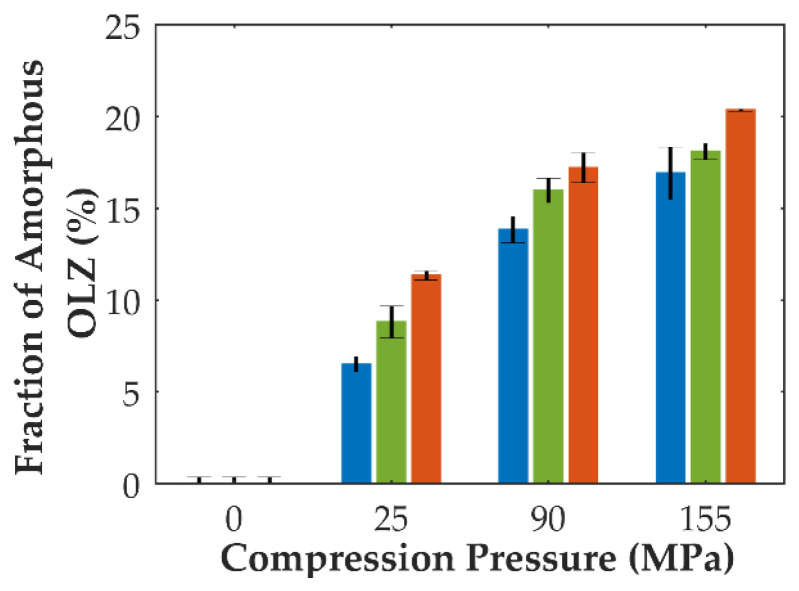
Predicted fraction of amorphous OLZ (formulation B) according to the compaction pressure (in x axis) and dwell time applied to compacts (blue: 0 min, green: 2 min, and orange: 5 min). Predictions were made according to da Costa et al. [19].

In contrast, the application of pressure to mixtures containing crystalline OLZ and SAC (formulation B) suggested the amorphization of OLZ, according to the regression models. Indeed, higher fractions of amorphous OLZ could be obtained as a result of the application of higher compaction pressures and dwell times to the mixtures (Figure 3). Thus, the energy supplied to the powder bed during tableting proved to promote the conversion of crystalline into amorphous OLZ (up to 20% at 155 MPa and 5 min dwell time).

Pressure-induced amorphization has been largely described in the literature for minerals or metallic crystals [56,57,58]. According to Yamanaka et al. [59], throughout compaction, a reversible amorphization of materials may be observed. This is associated with the instability of the dynamic lattice as a result of the application of shear and stress forces [59,60]. After pressure relief, the amorphous state may be reverted back into its crystalline counterpart [59], but for some materials, this amorphous state may be preserved even after the relaxation of the compacts. According to Ovid’ko [60], the plastic deformation occurring throughout compaction can be interpreted as the motion of defects within the crystalline arrangement of solids promoting the creation of a new nucleus different from those present in the parent lattice structure [59,60]. In pharmaceutics, pressure-induced amorphization has been barely described [19,20,61,62]. Huang et al. [20] elucidated the principles behind the conversion of crystalline into amorphous forms of posaconazole during compaction at pressures as low as 50 MPa. At higher compaction pressures, a nonlinear increase in the fraction of the amorphous drug was attained due to the high mechanical and thermal stresses imposed on the systems. Moreover, shear stress in particle-particle and particle-tableting machine tools interfaces could promote plastic deformation, the breakage of bonds, dislocations and slip of planes, and lattice distortion of crystalline materials, which may cause defects in the crystalline habit. As plastic deformation shows a dependency on the contact time [63], a variation in the extent of amorphization of posaconazole at lower compaction speeds should be expected. However, when the compression rate was increased, the authors found that the extent of amorphization was unaffected, requiring further work on the evaluation of this parameter.

Accordingly, in the present study, the plastic deformation of materials seems to explain the in situ co-amorphization of OLZ. In this regard, the plasticity was assumed based on the characteristics of the materials of both the formulations considered and the tensile strength values of compacts. Dibasic calcium phosphate anhydrous is a common filler used in the manufacture of tablets that, throughout tableting, tends to fragment due to the stress forces applied [64]. The reduced fraction of dibasic calcium phosphate anhydrous in formulation B, in contrast with formulation A, provided a system with higher plasticity. Further evidence of the higher plasticity in formulation B can be provided by the comparison of the tensile strength of the tablets obtained (Table 4). According to Thakral et al. [65], compacts containing excipients with high plasticity tend to result in the fabrication of tablets presenting higher tensile strength values. Formulations containing crystalline OLZ and SAC (formulation B, Table 4) presented higher tensile strengths than tablets made of crystalline OLZ (formulation A). This can ultimately be used to prove the higher plasticity and adhesion of the materials present in formulation B compared to formulation A. The increased plasticity of formulation B, together with the presence of SAC as a co-former, can promote the amorphization of OLZ during tableting. It is worth pointing out the parallelism between this study and that by Huang et al. [20], both of which report the same extent of amorphization during tableting (20–25%).

In fact, by increasing the dwell time applied to compacts, increased amorphization of mixtures containing crystalline OLZ and SAC was observed (Figure 3). For longer dwell times, plastic yielding seems to be favored resulting in a greater extent of amorphization. This pressure-induced amorphization presents inherent advantages compared to traditional amorphization techniques. In fact, the preparation of CAMs prior to the manufacture of tablets can be discarded, eliminating the stability issues of the feeding material and requiring a smaller number of unit operations, thus reducing production costs.

Stability studies were run under shelf storage conditions (23 °C/65% of relative humidity) to investigate the extent of recrystallization of OLZ-CAMs. XRPD diffractograms of samples stored for 3 years have shown that OLZ was maintained in the CAM system with SAC since no noticeable modifications in diffractograms were detected compared to fresh samples (data not shown). Therefore, the manufacture of tablets containing OLZ in a long-lasting, more soluble amorphous form is highlighted in this work.

### 3.4. Tableting

Tablets containing the model drug OLZ are available in the market in multiple drug dosages (2.5–20.0 mg per tablet). The 66.5 mg tablets prepared in the present work correspond to 20 mg of OLZ. Below the compaction pressure of 25 MPa, tablets crushed upon handling, hampering characterization. Above 155 MPa, a stabilization of the tensile strength of compacts was observed, anticipating no further significant consolidation of particles at higher pressures.

Tablets’ tensile strength is a measure of the pressure required to fracture a compact and thus elucidates the resistance of the dosage form. The determination of the tensile strength is particularly important for tablets since they need to present optimal strength to simultaneously enable further processing (e.g., coating and packaging) and facilitate the release of the drug [66]. In this regard, tablets containing amorphous OLZ and OLZ-CAM showed higher tensile strength values than tablets containing their crystalline counterpart (Table 4). This may indicate that the interactions established between the particles present in mixtures containing the amorphous OLZ and the OLZ-CAM were more intense than in mixtures containing the crystalline forms of the drug. Cespi et al. [67] evaluated the effect of temperature on the tablet properties of four compounds, namely microcrystalline cellulose, dihydrate dicalcium phosphate, ammonium methacrylate copolymer type B, and poly(ethylene oxide) (600,000 Da). Microcrystalline cellulose and dihydrate dibasic calcium phosphate showed no thermal transitions in the temperature range of 20–150 °C. By tableting these substances at different temperatures, the authors found that these substances showed to be slightly or not affected by changes in temperature. Inversely, tablets containing ammonium methacrylate copolymer type B or poly(ethylene oxide) presented different hardness values according to the temperature of the powder bed during tableting. This was justified by the authors due to the thermal transitions at low temperatures that these substances presented. Poly(ethylene oxide) showed a melting temperature in the range of 50–75 °C, whereas the ammonium methacrylate copolymer type B showed a glass transition at 50–60 °C. Taking this into consideration, the increase in the temperature bed during tableting of mixtures containing amorphous OLZ and OLZ-CAM to values above or closer to the Tg may have promoted the softening of amorphous OLZ particles, which in turn favored the establishment of interparticular bonds. Further evidence of the increase in the temperature of the powder bed to values above the Tg may be provided based on the work of compaction.

The work of compaction of blends indicated that formulations containing amorphous OLZ and OLZ-CAM retained more energy in the powder bed at the desired compaction pressure (Table 4). The work of compaction represents the total energy involved in the compaction of a powder at the desired compaction pressure and includes the energy transferred to particle rearrangement, elastic and plastic deformation, brittle fracture, and formation of bonds between the particles present in the powder bed [68]. The conversion of the rigid to the rubbery forms of the amorphous OLZ and the OLZ-CAM associated with the glass transition is an endothermic event, and thus, the sample needs to absorb energy through the transition. Therefore, the additional work of compaction needed to produce the tablets containing both amorphous OLZ and OLZ-CAM may be associated with the energy provided to the glass transition of materials.

The ejection force of tablets is indicative of the force required to remove the tablet from the tableting machine [69] and is related to lubrication issues. Measurement of the ejection force has failed to show statistical differences (*p* > 0.05) between tablets containing pure crystalline or amorphous OLZ, suggesting a similar extent of residual die wall stress. Inversely, tablets made of OLZ-CAM showed significantly higher values than their crystalline counterpart. The high ejection force for tablets containing the OLZ-CAM is likely to result in the appearance of defects in tablets, such as capping or sticking [70]. Thus, for formulations containing CAMs, the evaluation of the lubrication of powders should be reinforced to minimize the appearance of such defects.

Disintegration testing in demineralized water showed that tablets containing amorphous OLZ and OLZ-CAM required longer times to disintegrate than those containing their crystalline counterparts (Table 4). This could be assigned to the mechanism controlling the disintegration of tablets. When immersed in water, tablets containing the amorphous forms of OLZ have shown a slow erosion from the surface. It may be hypothesized that during immersion in water, a viscous gel at the surface of the tablet may be formed as a result of the diffusion of the amorphous form of OLZ, which in turn delayed the disintegration of tablets. This erosion, supported by previous observations [28], resulted in longer disintegration times for tablets containing CAMs. Inversely, tablets containing crystalline OLZ, or in a mixture with SAC, have rapidly disintegrated into small particles.

**Figure 4:**
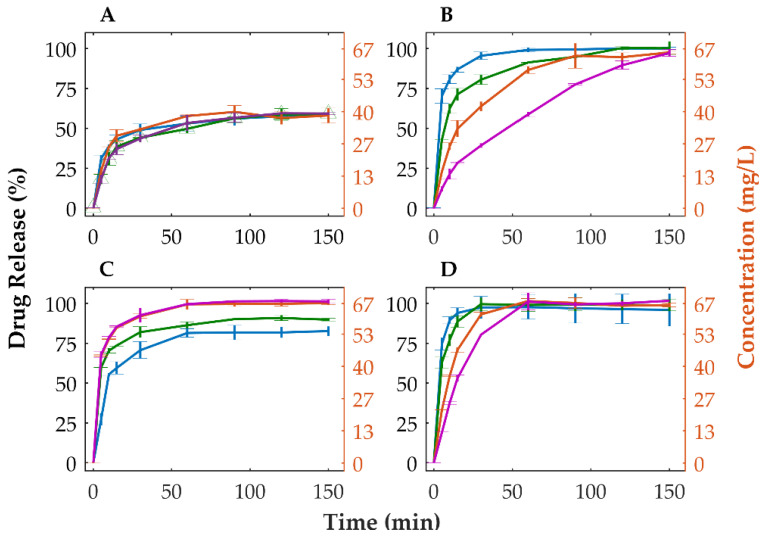
Dissolution profiles of crystalline (**A**) and amorphous OLZ (**B**) in formulation A and crystalline (**C**) and co-amorphous OLZ (**D**) in formulation B. [0 MPa (physical mixtures, blue), 25 MPa (green), 90 MPa (orange) and 155 MPa (purple) tablets].

Dissolution studies were performed to assess the extent and rate of release of OLZ from powdered physical mixtures and tablets. Powdered physical mixture and tablets made of crystalline OLZ (in formulation A) showed that approximately 58.9% (equivalent to 39.3 mg/L) of the drug was dissolved after 150 min (Figure 4A). Inversely, the amorphous forms of OLZ (i.e., amorphous and CAMs) released higher amounts of the drug (>95%) regardless of the compaction pressure applied to compacts, without evidence of recrystallization of OLZ (Figure 4B). Furthermore, dissolution tests performed on marketed tablets showed the same extent of drug release, at approximately 58.4%, most likely due to the utilization of the polymorphic form I of OLZ as feedstock material in the manufacture of tablets (Figure S1).

Concomitantly, powdered physical mixtures containing amorphous OLZ and OLZ-CAM presented a minimum of 2-fold increase in the quantity of OLZ dissolved at 15 min compared to the physical mixture containing crystalline OLZ. Inversely, when compacted, tablets containing amorphous OLZ and OLZ-CAM presented longer disintegration times (Table 4) which negatively affected the release of OLZ from tablets (Figure 4B,D). Particularly, tablets made of OLZ-CAM released a lower fraction (controlled release) of OLZ in the first minutes of the dissolution study due to the erosion of tablets, as described previously [71]. The slower release of the drug in the amorphous (Figure 4B) or CAM (Figure 4D) state, observed for tablets compacted at higher compaction pressures, was aligned with the longer disintegration time observed for those tablets (Table 4). Noteworthy is that the incorporation of 5% of croscarmellose sodium in the formulation containing the OLZ-CAM enabled a faster disintegration of the tablet (from 420.0 ± 87.2 to 49.5 ± 10.6 s, for a compaction pressure of 90 MPa, and from 1364.5 ± 12.02 to 157.3 ± 8.6 s, for a compaction pressure of 155 MPa) and the reduction in the time required to release 80% of OLZ (from 21.2 ± 1.0 min to 11.8 ± 0.5 min, for a compaction pressure of 90 MPa, and from 29.8 ± 0.2 min to 19.0 ± 1.2 min, for a compaction pressure of 155 MPa). These values are well below the dissolution acceptance criteria for immediate release in oral solid dosage forms (80% of drug release in 30 min [72]). The incorporation of croscarmellose sodium (5%), a polymeric plastic material, in the formulation containing OLZ-CAM was achieved by reducing the fraction of dibasic calcium phosphate anhydrous to 22%. The selective reduction in this excipient was considered since the tablets manufactured presented a smaller variation (<2%) of the tensile strength compared to those without croscarmellose sodium. On the other hand, tablets in which a proportional modification of all excipients was made presented a higher variation (>7%) of the tensile strength compared to tablets without croscarmellose sodium, thus most likely affecting the disintegration of tablets. Due to the small variation, the impact of the tensile strength on the disintegration time was disregarded. Thus, the higher dissolution rate, together with the higher solubility of tablets containing OLZ-CAM, anticipate a higher bioavailability of the drug.

Tablets containing the crystalline mixture of OLZ and SAC revealed a higher dissolution rate at the beginning of the test as a result of the rapid disintegration of tablets compared to tablets containing the CAM (Figure 4C,D). Among the compression pressures used to compact the mixtures, the tablets produced at a compression pressure of 25 MPa presented a lower release rate of the drug than those prepared at 90 or 155 MPa, which was expected due to the lower extent of amorphization of the drug (Figure 3). In this respect, the maximum pressure applied to the blend (155 MPa) would produce tablets with the maximum dissolution rate, which was not the case (Figure 4C). On the other hand, at the end of the dissolution test, the fraction of OLZ released from tablets has shown to increase accordingly with the compaction pressure applied to compacts. This is in line with the previous observations concerning the pressure-induced amorphization in which a higher fraction of amorphous material could be achieved due to the application of higher compaction pressure to the compacts (Figure 3). Noteworthy is that above 90 MPa, the total quantity of OLZ present in the dissolution vessels was dissolved in the buffer, suggesting that the solubility of OLZ was enhanced to values higher than 66.7 mg/L. In contrast, the lower extent of amorphization of OLZ, achieved by the application of a compression pressure of 25 MPa (compared to 90 and 155 MPa), although enhancing the solubility of the drug, it was insufficient to saturate the dissolution media. Consequently, one may assume that a pressure threshold (between 25 and 90 MPa) exists, reflecting the minimum extent of OLZ amorphization required to saturate the dissolution media (66.7 mg/L), predominating over the opposite effect of pressure on the release of OLZ. On the contrary, when the pressure was increased above 90 MPa, the pressure became the prime effect due to the decrease in porosity and increase in interparticular bonds within the tablet structure. Thus, the dissolution profile of OLZ for tablets containing the mixture of crystalline OLZ and SAC can ultimately be used to validate the in situ amorphization of OLZ throughout compaction.

## 4. Conclusions

In this work, the use of amorphous OLZ and OLZ-CAM in the production of tablets was investigated and has proved to enhance the extent of drug release, according to dissolution testing. The cohesiveness of OLZ-CAM powders was increased, with a negative impact on flowability, as reflected by the angle of repose and Carr’s index measurements. Poor flowability may result in the inadequate filling of the tableting die cavities, and thus, formulation materials and/or strategies that promote flow should be considered to guarantee the production of dosage forms with the required quality attributes.

The use of validated recrystallization predictive models based on XRPD, FTIR, and NIR spectroscopy data indicated that amorphous OLZ and OLZ-CAM were stable to the application of different compaction pressures and dwell times. Additionally, during tableting, a pressure-induced amorphization of OLZ was observed when the drug was incorporated in formulations containing SAC. The extent of amorphization increased with the compaction pressure and dwell time imposed on manufacturing the compacts, suggesting that the plastic deformation of materials may promote the amorphization of OLZ and, thus, enhance OLZ solubility in tablets, particularly when these are obtained at high compaction pressures and dwell times.

Tablets containing OLZ-CAM showed enhanced tensile strengths because they retained more energy at the defined pressure than physical mixtures of OLZ:SAC due to the higher cohesiveness and softness of OLZ-CAM during tableting. Concomitantly, a higher disintegration time was observed, anticipating the lower release of the drug in the first minutes of dissolution. This negative impact on drug release may be circumvented by the use of superdisintegrants (e.g., croscarmellose) or the application of lower compaction pressures to the powder bed.

## Figures and Tables

**Table 1:** Composition (%) of formulations A and B used for tableting.

Substance	A	B
OLZ	30	30
SAC	0	18
Dibasic calcium phosphate anhydrous	45	27 *
Microcrystalline cellulose	20	20
Polyvinylpyrrolidone	5	5

* in formulations containing sodium croscarmellose (5%), the fraction of dibasic calcium phosphate anhydrous was reduced to 22%.

**Table 2:** Cohesion index, cake strength, and angle of repose of pure OLZ and physical mixtures of OLZ and SAC in both crystalline and (co-)amorphous forms.

	Cohesion Index (mm)	Cake Strength (g)	Angle of Repose (°)
**OLZ**			
Crystalline	14.3 ± 0.6	174.7 ± 3.4	51.7 ± 0.1
Amorphous	15.8 ± 0.3 *	181.4 ± 9.9	52.8 ± 0.3 *
**OLZ:SAC**			
Crystalline	12.7 ± 0.4	159.6 ± 6.9	51.0 ± 0.9
Co-amorphous	25.2 ± 1.2 **	180.7 ± 5.9 *	54.6 ± 1.7 *

* *p* < 0.05 and ** *p* < 0.01 versus the respective crystalline counterpart.

**Table 3:** Pycnometric density and Carr’s index of crystalline and amorphous OLZ, as pure material or in physical mixture with SAC.

	True Density (g/cm^3^)	Carr’s Index
**OLZ**		
Crystalline	1.3053 ± 0.0022	39.7 ± 0.5
Amorphous	1.2764 ± 0.0014 **	41.3 ± 0.6 *
**OLZ:SAC**		
Crystalline	1.3897 ± 0.0017	34.0 ± 1.0
Co-amorphous	1.3501 ± 0.0058 **	36.7 ± 1.1 *

* *p* < 0.05 and ** *p* < 0.01 versus the respective crystalline counterpart.

**Table 4:** Properties of tablets (tensile strength, disintegration time, work of compaction, and ejection force) produced under different compaction pressures (considering both crystalline and (co-)amorphous OLZ incorporated in formulations A and B).

	Tensile Strength (MPa)			Disintegration Time (s)			Work of Compaction (J)			Ejection Force (kN)		
Compaction Pressure (MPa)	25	90	155	25	90	155	25	90	155	25	90	155
**Formulation A**												
Crystalline	0.12 ± 0.00	0.95 ± 0.01	1.64 ± 0.04	31.0 ± 2.7	26.2 ± 3.3	64.7 ± 3.1	0.184 ± 0.007	0.839 ± 0.018	1.357 ± 0.011	0.014 ± 0.001	0.093 ± 0.010	0.142 ± 0.014
Amorphous	0.29 ± 0.00 **	1.14 ± 0.02 **	2.11 ± 0.08 **	686.3 ± 63.2 **	2470.7 ± 45.0 **	7131.0 ± 201.06 **	0.212 ± 0.013 *	0.890 ± 0.028 **	1.422 ± 0.018 **	0.018 ± 0.002	0.089 ± 0.007	0.133 ± 0.012
**Formulation B**												
Crystalline	0.16 ± 0.01	1.01 ± 0.05	1.84 ± 0.04	56.0 ± 3.6	48.7 ± 4.5	63.7 ± 5.9	0.192 ± 0.005	0.902 ± 0.028	1.382 ± 0.020	0.028 ± 0.004	0.163 ± 0.009	0.288 ± 0.013
Co-amorphous	0.59 ± 0.01 **	1.97 ± 0.01 **	2.74 ± 0.05 **	167.0 ± 4.2 **	420.0 ± 87.2 **	1364.5 ± 12.02 **	0.250 ± 0.017 *	1.090 ± 0.034 **	1.754 ± 0.025 **	0.054 ± 0.014	0.234 ± 0.026 *	0.433 ± 0.041 *

* *p* < 0.05 and ** *p* < 0.01 versus the respective crystalline counterpart.

## Data Availability

Not applicable; the data presented is not publicly available.

## Supplementary Materials

The following supporting information can be downloaded at: https://www.mdpi.com/article/10.3390/pharmaceutics14081535/s1, Figure S1: Dissolution profile of OLZ present in marketed tablets.
