# Supplementary figures and images for: Downstream Processing of Amorphous and Co-Amorphous Olanzapine Powder Blends
Source: Pharmaceutics. 2022 Jul 23;14(8):1535. doi: 10.3390/pharmaceutics14081535 (PMC9332588; doi:10.3390/pharmaceutics14081535)

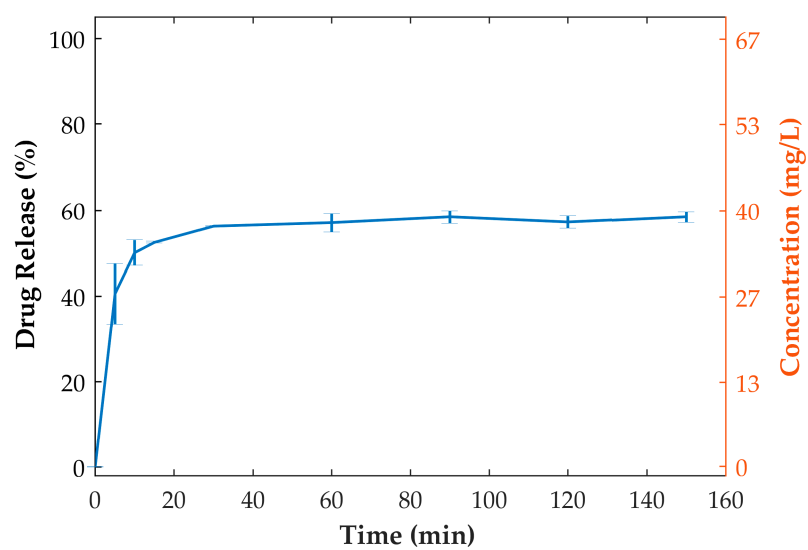

**Figure S1.** Dissolution profile of OLZ present in marketed tablets.

Supplement: Supplementary file 1 [file pharmaceutics-14-01535-s001.zip › pharmaceutics-1822154-supplementary.pdf]
